# A data quality assessment of the first four years of malaria reporting in the Senegal DHIS2, 2014–2017

**DOI:** 10.1186/s12913-021-07364-6

**Published:** 2022-01-02

**Authors:** Pierre Muhoza, Roger Tine, Adama Faye, Ibrahima Gaye, Scott L. Zeger, Abdoulaye Diaw, Alioune Badara Gueye, Almamy Malick Kante, Andrea Ruff, Melissa A. Marx

**Affiliations:** 1 Department of International Health, Johns Hopkins Bloomberg School of Public Health, 615 N. Wolfe Street, Baltimore, MD 21205 USA; 2 Département de Parasitologie, Faculté de Médecine, de Pharmacie et d’Odontologie, Université Cheikh Anta Diop de Dakar, Dakar, Senegal; 3 Institut de Santé et Développement, Université Cheikh Anta Diop de Dakar, Dakar, Senegal; 4 Department of Biostatistics, Johns Hopkins Bloomberg School of Public Health, 615 N. Wolfe Street, Baltimore, MD 21205 USA; 5 Direction de la Planification, de la Recherche et des Statistiques/ Division du Système d’Information Sanitaire et Sociale, Ministère de la Santé et de l’Action Sociale (MSAS), Dakar, Senegal; 6 Programme National de Lutte Contre le Paludisme, Ministère de la Santé et de l’Action Sociale (MSAS), Dakar, Senegal

## Abstract

**Background:**

As the global burden of malaria decreases, routine health information systems (RHIS) have become invaluable for monitoring progress towards elimination. The District Health Information System, version 2 (DHIS2) has been widely adopted across countries and is expected to increase the quality of reporting of RHIS. In this study, we evaluated the quality of reporting of key indicators of childhood malaria from January 2014 through December 2017, the first 4 years of DHIS2 implementation in Senegal.

**Methods:**

Monthly data on the number of confirmed and suspected malaria cases as well as tests done were extracted from the Senegal DHIS2. Reporting completeness was measured as the number of monthly reports received divided by the expected number of reports in a given year. Completeness of indicator data was measured as the percentage of non-missing indicator values. We used a quasi-Poisson model with natural cubic spline terms of month of reporting to impute values missing at the facility level. We used the imputed values to take into account the percentage of malaria cases that were missed due to lack of reporting. Consistency was measured as the absence of moderate and extreme outliers, internal consistency between related indicators, and consistency of indicators over time.

**Results:**

In contrast to public facilities of which 92.7% reported data in the DHIS2 system during the study period, only 15.3% of the private facilities used the reporting system. At the national level, completeness of facility reporting increased from 84.5% in 2014 to 97.5% in 2017. The percentage of expected malaria cases reported increased from 76.5% in 2014 to 94.7% in 2017. Over the study period, the percentage of malaria cases reported across all districts was on average 7.5% higher (*P* < 0.01) during the rainy season relative to the dry season. Reporting completeness rates were lower among hospitals compared to health centers and health posts. The incidence of moderate and extreme outlier values was 5.2 and 2.3%, respectively. The number of confirmed malaria cases increased by 15% whereas the numbers of suspected cases and tests conducted more than doubled from 2014 to 2017 likely due to a policy shift towards universal testing of pediatric febrile cases.

**Conclusions:**

The quality of reporting for malaria indicators in the Senegal DHIS2 has improved over time and the data are suitable for use to monitor progress in malaria programs, with an understanding of their limitations. Senegalese health authorities should maintain the focus on broader adoption of DHIS2 reporting by private facilities, the sustainability of district-level data quality reviews, facility-level supervision and feedback mechanisms at all levels of the health system.

**Supplementary Information:**

The online version contains supplementary material available at 10.1186/s12913-021-07364-6.

## Background

Progress in the fight against malaria has encouraged many malaria endemic countries including Senegal, to outline a vision for malaria elimination [1, 2]. Achieving success will likely require a combination of several interventions that are guided by a strong surveillance system [2]. Accordingly, the World Health Organization (WHO) has come to consider surveillance as a core intervention in malaria elimination settings [3]. In these contexts, routine health information systems (RHIS) are an important surveillance tool that can provide information that enables programs to monitor disease trends, health service utilization and access to interventions. The information collected should guide the decision-making around setting priorities and allocating resources for disease control. Highlighting the central role of RHIS in malaria control, the WHO recommended sustained investments in RHIS and surveillance as one of the three pillars of the 2015 strategy of reducing global malaria burden by 90% by 2030 [3].

The renewed interest in RHIS as a core component of malaria surveillance came at a time during which many countries were initiating efforts to strengthen their information systems using an open source web-based software platform known as the District Health Information System (DHIS2) [4]. This software enables the continuous collection, aggregation and visualization of health data at all levels of the health system. Given the dynamic and focal nature of malaria transmission, particularly in areas that have experienced sharp declines in morbidity, the DHIS2’s data management efficiency and granularity make it invaluable for meeting the surveillance data needs in low-resource countries. For example, countries such as Ghana, Kenya, Tanzania and Uganda are among some that use malaria DHIS2 data for their bulletins and reports [5–8].

Despite the significant investments made in the implementation of the DHIS2 platform in Senegal, concerns about the quality of DHIS2 data have contributed to low levels of use of the data for the reporting needs of the National Malaria Control Program (NMCP). DHIS2 was first introduced in Senegal in 2013 and initially piloted in the western regions of Dakar and Thies in 2014. Later that year, DHIS2 was rapidly deployed across the remaining 12 regions until the platform was declared the national RHIS in 2016 [9]. Between 2014 and 2017, the President’s Malaria Initiative and the Global Fund provided the Senegal Ministry of Health (MoH) with over $2.2 million to support activities related to the implementation and integration of DHIS2 across the 76 districts of the country [10, 11]. Although the NMCP and the MoH’s Division of Social and Health Information Systems (DSISS) are working closely to integrate the DHIS2 and the NMCP’s routine information system, the latter continues to be the primary source of malaria information [12].

As the NMCP continues to integrate its malaria surveillance activities into the DHIS2, progress in data reporting should be evaluated in order to identify any potential gaps for mitigation and opportunities for improvements. The aim of this study is to evaluate the quality of reporting of key indicators of childhood malaria during the first 4 years of DHIS2 implementation in Senegal. We present analyses guided by the WHO’s desk review of data quality [13] and we focus on the data quality dimensions of completeness and internal consistency.

## Methods

### Country profile

Located at the westernmost point of the African continent, Senegal is a low-income country of approximately 15.2 million inhabitants as of 2017 [14]. The percentage of the population living in urban areas is 46.5%. Children under 5 years old represent approximately 16.3% of the population [14].

Senegal’s climate is divided into two main seasons: the dry and rainy seasons. The rainy season lasts from July to October peaking during the months of August and September. The dry season runs from November through June [15].

**Fig. 1:**
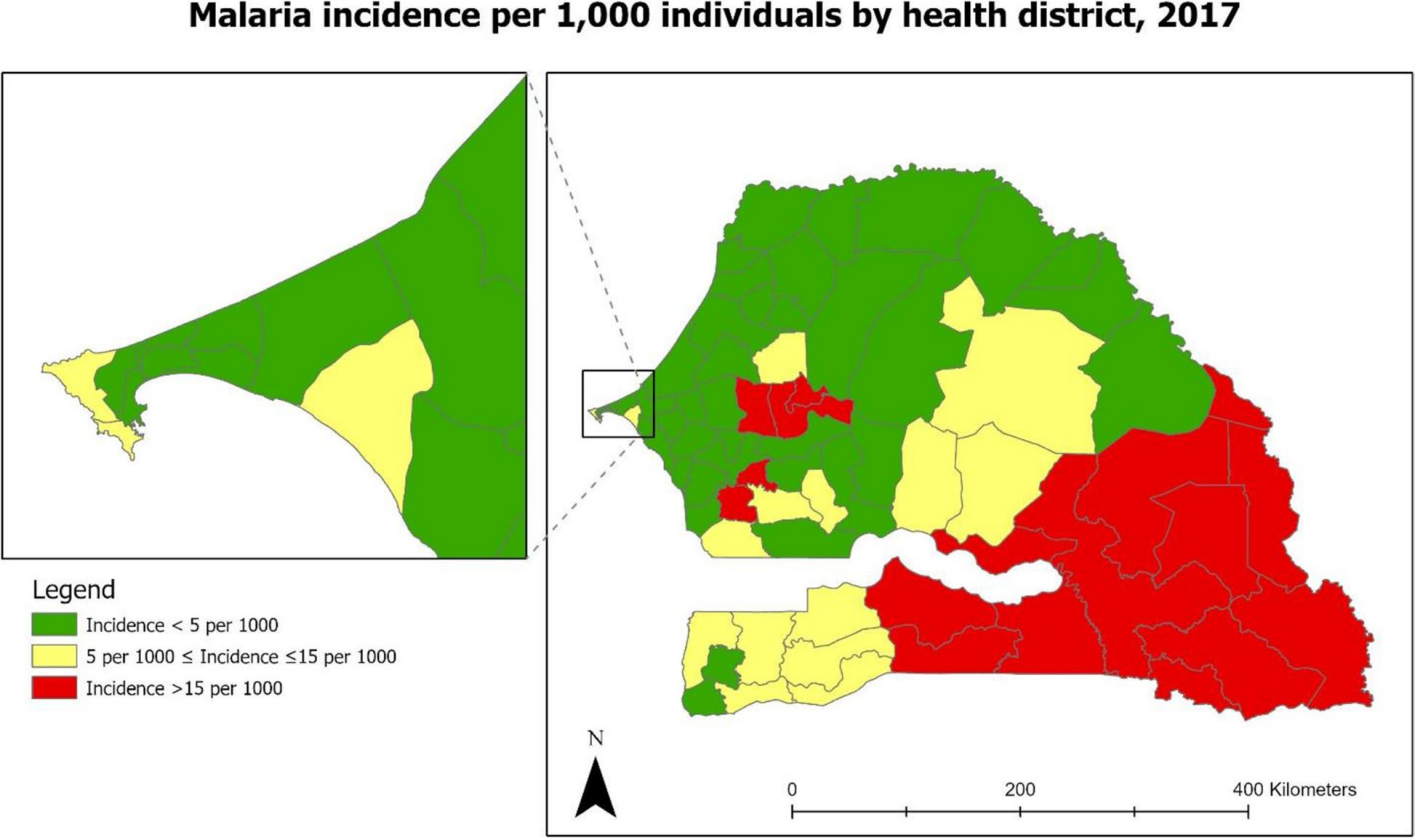
Map of Senegal showing the variation in malaria incidence across the 76 health districts. Figure adapted from the 2017 malaria epidemiological bulletin [17]

Transmission of malaria in Senegal is heterogeneous, often occurring within highly focalized hotspots across districts. Parasitemia among children under five ranges from 0% in the northern Saint-Louis region to 15.3% in the southeastern region of Kedougou [16]. The NMCP uses routinely collected malaria incidence data to stratify the country’s 76 districts into 3 zones according to the annual malaria incidence. In this study, we used the NMCP’s 2017 stratification of districts by malaria transmission zone (Fig. 1) [17]. In 2017, there were 42 districts in the low transmission zone where malaria incidence was < 5 cases per 1000 inhabitants [17]. There were 17 districts each in the moderate and high transmission zones where the incidence was 5 to 15 and over 15 cases per 1000 inhabitants, respectively [17]. The low, moderate and high malaria transmission zones respectively overlap with the country’s Sahelian, Sudano-Sahelian and Sudano-Guinean bioclimatic zones. Transmission is mainly due to the *Plasmodium falciparum* parasite accounting for at least 99% of malaria infections with the other species, *Plasmodium malariae, Plasmodium ovale, and Plasmodium vivax* representing the remaining 1% [18, 19]. The most common malaria vectors are the *Anopheles gambiae, Anopheles arabiensis* and *Anopheles funestus* species of mosquitoes [18].

In most of the country, the malaria surveillance system is passive and thus relies on the routine notification of cases by healthcare facilities. In the northern districts where transmission is low, the NMCP has instituted reactive case detection strategies since 2011. Starting from 2016, the NMCP employed a range of malaria control strategies that included mass drug administration (MDA), focal test and treat (FTAT) and focal screen test and treat (FSTAT) for select districts in the north that reached the pre-elimination threshold (defined as annual incidence < 5 cases per 1000) [18]. Infections identified through facility-based passive case detection are reported to the district-level health teams, which then initiate investigations to identify secondary cases at the community level [20].

Each health district has at least one public health center and a number of public health posts that are staffed by chief nurses and sometimes midwives [21]. Health posts provide primary care whereas health centers provide first-level referrals and limited hospitalization services. The referral hospitals provide specialized health care services.

At the base of the Senegalese health system are health huts, which are not recognized as a formal part of the health system. These health huts nonetheless cover approximately 50% of the population [21]. Each health hut includes a trained community health worker (CHW) who is regularly supervised by the chief nurse of the health post and also includes a structure through which essential commodities for health services can be obtained by the CHW. Among remote populations in villages situated at least 5 km from a facility and not served by a health hut, active case finding and community-based management of malaria are conducted by a specific type of CHW known as DSDOM (*dispensateur de soins à domicile*). These activities are conducted as part of the NMCP’s strategy of home-based management of malaria commonly known as PECADOM (*prise en charge à domicile*) [22].

Diagnosis of malaria is almost exclusively conducted using rapid diagnostic tests (RDTs) and their availability has remained high in Senegal. RDT coverage across all health facilities was stable ranging between 94% in 2014 to 90% in 2017, with almost all public facilities reporting RDT supplies during the study period [23–26]. While microscopic diagnosis of malaria is available in most hospitals and health centers, it is rare to find in health posts.

According to the 2017 Senegal Service Provision Assessment (SPA) survey, the country’s public health sector consisted of 35 referral hospitals, 100 health centers, 1458 health posts and 2464 health huts [21]. In addition to the public health facilities above, Senegal also has a vibrant private health sector comprising of 359 medical offices (*cabinets medicaux)*, 443 paramedical offices *(cabinets paramedicaux)*, 2 referral hospitals, 115 clinics, 111 health posts and 1013 pharmacies [21, 27].

Malaria-related service delivery is largely delivered by the public sector although the private sector is increasingly playing a major role, particularly in urbanized areas [16, 27]. Both public and private facilities are expected to submit monthly surveillance reports to the district and central levels. Though public facilities are generally known to report at higher levels compared to private facilities, the precise proportions of facilities that submit reports remain unknown [27].

### Malaria reporting in Senegal

Uncomplicated malaria cases are typically managed at the community or health post levels whereas more complicated cases are referred to higher levels of the health system. Starting with paper-based data collection at the health hut or DSDOM level, community routine data flow up to health posts, where they are then combined with facility data and compiled into a paper-based report summarizing services delivered by the health facility during the month. Prior to 2016, the report would be sent to the district level where data would be reviewed by the health management team. From that point, the data would be summarized into an Excel-based template provided by the NMCP and also entered into the DHIS2 system. To improve the efficiency of data collection processes, data entry tasks into the DHIS2 system were shifted from the district health management team to the individual chief nurses of health facilities. Since 2016, chief nurses receive training and login credentials enabling them to enter data directly into the DHIS2 system on a monthly basis. The chief nurses are required to enter into the system the number of services delivered and report a value of “zero” in instances where no services have been provided. In such cases, however, chief nurses sometimes choose to leave the data field blank instead of entering a value of zero as required. This makes it impossible to distinguish true missing values (i.e., no data reported) from ‘*zero*’ values (i.e., no events captured).

On quarterly basis, the Chief District Medical Officer prepares a synthesized report that is sent simultaneously to the regional and central levels. Theoretically, the district health management team is required to organize quarterly data review meetings with representatives of each health post in the district to validate data in the DHIS2 and the NMCP Excel spreadsheets using facility reports. Nonetheless, it is unknown to what extent this procedure is followed consistently.

### Assembling DHIS2 malaria case reports (January 2014 – December 2017)

The analyses in this study focused on three indicators that are programmatically important in settings like Senegal where malaria transmission is heterogeneous [28]. These indicators included the total number of confirmed and suspected malaria cases among children under the age of 5 and rapid diagnostic tests (RDTs) performed. The indicators are reported by both primary and referral health facilities and together, they enable the calculation of malaria case incidence, test positivity rate, testing rates. Case incidence is the primary recommended impact indicator for both moderate- and low-transmission settings, whereas the test positivity rate is key for malaria surveillance in moderate to high transmission settings [28, 29]. The testing rate provides information on the extent of parasitological confirmation among those clinically diagnosed with malaria which can be useful in the interpretation of the previous indicators [28]. In the analyses of these indicators, we took into account service delivery in both the outpatient and community settings.

Data for the indicators reported by health facilities were extracted from the DHIS2 online database in October 2019. Monthly data representing January 2014 and December 2017 were extracted separately for each district, disaggregated by health facility and arranged chronologically. The assembled district data sets were merged into one database for the country.

### Data analysis

#### Completeness of reported data

The completeness of facility reporting was measured as the number of monthly reports received divided by the expected number of reports in a given year (12 months x number of health facilities reporting that year). Districts with reporting completeness rates below 80% were considered to have poor reporting [13]. Completeness of indicator data was measured as the percentage of non-missing values across the three indicators. This was calculated at the national level by first summing all non-missing indicator values and dividing by the expected number of values (12 months × 76 districts × 3 indicators).

To calculate the percentage of expected cases reported, we first used reported facility data to interpolate missing values between months. We used a quasi-Poisson model to calculate a smoothed curve of the expected monthly number of confirmed malaria cases from each facility. Given the observed overdispersion in the case count data, the quasi-Poisson model was chosen as it assumes that the variance is a function of the mean [30, 31]. Since seasonality is a dominant pattern in monthly case count data, we controlled for it using natural cubic spline terms [32–34]. Splines are smoothing functions that can control for confounding due to season effects and secular trends over a given time period using curves joined at time points called knots [34]. Based on the seasonal changes observed in the case count data, we placed three internal knots at February, June, and October of each year to allow flexibility in the curve at each of these points [33]. The season effect was defined by the time between January and December of each year whereas the secular trend was defined by the uninterrupted time between January 2014 and December 2017. The model’s predictors thus were the facility and an interaction term of the season and secular trend effects to allow smooth changes in the seasonal wave over the study period. Missing case count data were replaced with values predicted from the specified model. The ratio of observed case counts and observed case counts with the imputed values was then calculated. This ratio measures the percentage of expected cases that are actually reported. To test the hypothesis that reporting completeness differs by season, we performed two-sample t tests for equal means comparing the monthly values of the percentage of cases reported or percentage of facilities reporting during the dry versus rainy seasons.

#### Internal consistency of reported data

The internal consistency of the reported data was evaluated using two approaches. In the first approach, we identified moderate and extreme outliers. Moderate outliers were defined as monthly values that deviated from the district’s monthly mean value of the indicator by at least 2 standard deviations. Extreme outliers deviated by at least 3 standard deviations. The second approach involves a trend analysis to examine the plausibility of reported results for the selected malaria indicator based on the history of reporting for the indicator. Starting from 2017 and working backwards to 2014, the annual indicator value was compared to the mean of the three preceding years combined. This ratio was calculated at national and district levels. Districts with at least a 33% difference between their ratio and the national ratio were identified as reporting inconsistently. 33% is the threshold recommended by the WHO desk review of routine data quality, a standardized series of checks used to assess the quality of RHIS data [13]. Furthermore, this threshold has been previously used by others [35].

Stata version 14.2 (StataCorp LLC, College Station, TX) was used for all analyses described in this study [36].

## Results

### Description of health facilities analyzed

**Fig. 2:**
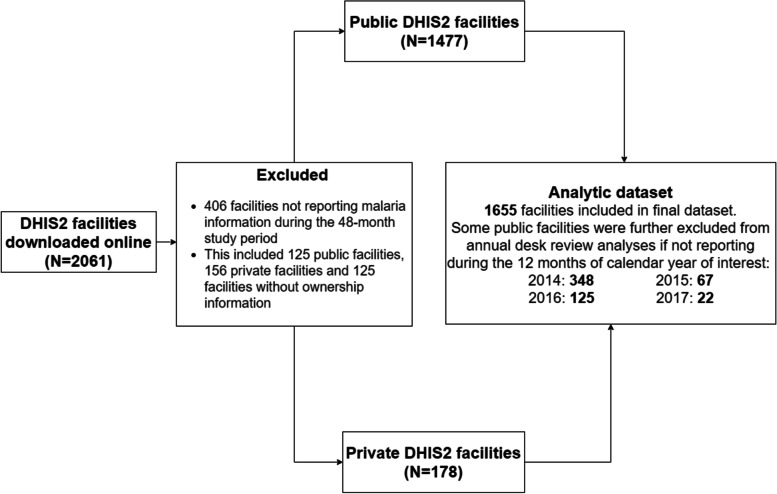
Flow diagram of database processing to generate the data set used in this study

The dataset assembled from the Senegal DHIS2 data contained 2061 facilities. During data cleaning, 406 (19.7%) facilities not reporting on any malaria indicators (or any of the core indicators recommended by the WHO desk review framework) were excluded from analysis. Of the excluded facilities, 125 were public, 156 were private and 125 did not have ownership information (Fig. 2). Of the 125 excluded public facilities, 30 (24%) were from the Dakar region. Across the three core indicators examined in this study, 34.6% of the data values spanning the study period were missing from the 2061 facilities configured in the DHIS2.

The public sector was well-represented in the DHIS2 with 1477 (92.7%) of the 1593 public facilities enumerated by the 2017 Senegal SPA survey reporting during the study period. On the other hand, only 178 (15.3%) of the 1163 private facilities enumerated by the same survey were found to be reporting data in the DHIS2 over the study period (Table 1). Notably, only 19 (2.4%) of the 802 private medical offices enumerated by the SPA survey reported during the study period. Given the low levels of private sector representation in the DHIS2, we henceforth report results from public facilities only. Across the three core indicators, 18.7% of the data values were missing from the public facilities included in the analysis.

**Table 1:** Description of health facilities in analytic sample by type and sector

Facility type	Managing authority		
	Public n (%)	Private^**a**^ n (%)	Total n (%)
**Medical offices**	0 (0.0)	19 (100.0)	19 (100.0)
**Clinics/Dispensaries**	0 (0.0)	34 (100.0)	34 (100.0)
**Health posts**	1336 (91.8)	120 (8.2)	1456 (100.0)
**Health Centers**	103 (100.0)	0 (0.0)	103 (100.0)
**Hospitals**	38 (88.4)	5 (11.6)	43 (100.0)
**Total**	**1477 (100.0)**	**178 (10.8)**	**1655 (100.0)**

^a^ Private facilities are excluded from further analyses given their low numbers in the dataset

As expected, health posts represented the vast majority (87.9%) of the facilities configured in the DHIS2 system (Table 1). Public health posts alone accounted for 76.1 and 84.5% of the reported suspected and confirmed cases over the study period, at average of rates of 19.8 and 2.8 cases per facility per month, respectively. Of the 1458 public health posts enumerated by the 2017 SPA survey, 1336 (91.0%) were represented in the DHIS2 and reported at least once during the study period. Similarly, we found 103 public health centers and 38 hospitals represented in the DHIS2 system compared to the 100 and 35 respectively enumerated by the 2017 SPA survey. The health centers accounted for 20.8% and 13.9% of the suspected and confirmed cases, at average rates of 67.3 and 5.8 cases per facility per month, respectively. Hospitals accounted for 2.8% and 1.6% of the suspected and confirmed cases, respectively, at average rates of 31.2 and 2.9 cases per facility per month. The distribution by type and over time of the public facilities analyzed in this study is provided in Table 2 whereas the stratification across malaria transmission zones is provided in supplementary Table 1.

**Table 2:** Description of public facilities reporting malaria data in DHIS2 by year

Facility type	2014 n (%)	2015 n (%)	2016 n (%)	2017 n (%)
**Health posts**	1038 (92.0)	1214 (89.8)	1272 (90.2)	1318 (90.6)
**Health centers**	84 (7.4)	103 (7.6)	102 (7.2)	102 (7.0)
**Hospitals**	7 (0.6)	35 (2.6)	36 (2.6)	35 (2.4)
**Total**	**1129 (100.0)**	**1352 (100.0)**	**1410 (100.0)**	**1455 (100.0)**

Among the public health posts included in the analysis, the number of facilities reporting community-based malaria data in addition to the facility-based data increased from 700 (67.%) to 1189 (90.2%) (supplementary Table 2).

### Reporting completeness in public health facilities

Completeness of facility reporting increased from 2014 with 84.5% of facilities reporting in the DHIS2 to 97.5% in 2017 (Table 3). The number of districts with facility reporting rates below 80% decreased from 20 in 2014 to 0 in 2017. With the exception of 2016, we found that the average percentage of cases reported across districts was higher during the rainy season compared to the dry season (Table 3). Combining all annual data, we found the average district percentage of cases reported during the rainy season to be 7.5% higher (*P* < 0.01) relative to the dry season. Similarly, the district percentage of facilities submitting a monthly report was found to be 1.3% higher during the rainy season compared to the dry season although the difference was not statistically significant (*P* = 0.07). Annual comparisons are presented in Table 3.

**Table 3:** Examination of outliers and internal consistency between indicators (2014–2017)

	2014	2015	2016	2017
**Percentage of values that are moderate outliers** ^**a**^	5.6	5.6	5.2	4.4
**Percentage of values that are extreme outliers** ^**a**^	2.2	2.5	2.4	2.1
**Number of districts reporting > 5% moderate outlier monthly values**	51	55	47	28
**Number of districts reporting > 5% extreme outlier monthly values**	1	2	4	1
**Percentage of events where confirmed cases > tested cases**	0.06	0.03	0.01	0.01

^a^ Numerator = sum of occurrences of outliers [± 2(3) SD relative to the mean of reported values] over the 12 months for the 3 indicators; Denominator = (number of health facilities multiplied by 12 months)

Analysis of completeness rates by malaria transmission zones showed improvements across all zones (Table 3). During the study period, an average of 90.4% of the expected malaria cases were reported across districts in the moderate transmission zone compared to 82.1 and 87.4% in the low and high transmission zones, respectively. During the same period, the average district-level reporting completeness rates were 91.6%, 93.8% and 89.8% for the low, moderate and high transmission zones, respectively.

We found general improvements in the completeness of reporting of the malaria indicators across all types of public facilities (Table 4). Across all malaria transmission zones, on average health centers and health posts reported at roughly similar levels during the study period. On the other hand, hospitals were on average less likely to submit a monthly report and submitted data with higher levels of missing values than health posts and health centers (Table 4). Across all facility types, we generally found that facilities in the moderate transmission zone had better completeness rates compared to facilities from other zones with the facilities from the high malaria transmission zone having the lowest completeness rates on average (Table 4).

**Table 4:** Consistency over time: national ratio of total number of events in 2017 to mean number of events in preceding 3 years

Indicator	2017	
	Ratio	Number and proportion of districts with 33% difference between their ratio and national ratio
**Number of confirmed cases**	1.15	14 (18.4%)
**Number of suspected cases**	2.22	8 (10.5%)
**Number of RDTs done**	2.24	9 (11.8%)

Across all indicators examined, zeros were common and unevenly distributed by transmission zone. During the 2014–2017 period, zeros accounted for 36.4%, 29.0% and 24.7% of the reported values in the low, moderate and high transmission zones, respectively. During the same period, 34.7% and 24.5% of values reported across the examined indicators occurred during the dry and rainy seasons, respectively. Supplementary Table 3 shows the occurrence of reported zero values by season across the examined indicators during each year of the study.

### Moderate and extreme outliers

At the national level, the percentage of moderate outliers decreased from 5.6% in 2014 to 4.4% in 2017 (Table 5). On the other hand, the percentage of extreme outliers fluctuated around an average of 2.3% during the study period. At the sub-national level, the number of districts reporting > 5% monthly values that were extreme outliers was generally low (Table 5). In comparison, the number of districts reporting > 5% monthly values that were moderate outliers was higher but generally decreased over time.

**Table 5:** DHIS2 reporting completeness among public facilities (2014–2017)

	2014**	2015	2016	2017
**National percentage of facilities submitting monthly reports**	85.4	92.8	96.1	97.5
**National percentage of expected cases reported**	76.5	83.1	94.1	94.7
**National percentage of non-missing indicator values**	84.8	92.5	95.7	97.3
**Districts with monthly facility reporting rate < 80%**	20	10	2	0
**Districts with non-missing indicator values < 80%**	21	10	3	0
Ratio of average district percentage of expected cases reported during rainy and dry seasons***	1.13 **(*P < 0.001*)**	1.04 **(*P = 0.03*)**	1.00 (*P = 0.83*)	1.13 **(*P < 0.0001*)**
Ratio of average district percentage of facilities reporting during rainy and dry seasons***	1.06 **(*P = 0.01*)**	1.00 (*P = 0.91*)	0.99 (*P = 0.71*)	1.00 (*P = 0.97*)
**Average district percentage of expected cases reported in the low transmission zone**	76.7	84.5	82.0	85.0
**Average district percentage of expected cases reported in the moderate transmission zone**	78.9	90.2	98.2	94.2
**Average district percentage of expected cases reported in the high transmission zone**	73.6	87.9	96.7	91.5
**Average district percentage of facilities reporting in the low transmission zone**	85.4	89.3	94.6	96.9
**Average district percentage of facilities reporting in the moderate transmission zone**	84.1	93.9	98.7	98.3
**Average district percentage of facilities reporting in the high transmission zone**	77.1	91.2	96.8	93.9

^**^ Analyses do not include data from 5 districts from the regions of Fatick (moderate transmission zone) and Matam (low transmission zone) that did not report malaria data in the DHIS2 throughout 2014

***P values result from a two-sample t-test assuming equal variances comparing district percentage of cases reported during rainy versus dry seasons. Bolded values are statistically significant (< 0.05)

### Consistency over time

Comparing 2017 national health events to the mean of the three preceding years, the number of confirmed cases increased by 15%, whereas the numbers for suspected cases and RDTs done both more than doubled. Table 6 presents comparisons of the national ratios to district ratios for the selected indicators. The majority of districts have ratios that are consistent with national ratios (within 33%) suggesting that the consistency of indicators has improved over time.

**Table 6:** DHIS2 reporting completeness by facility type in the public sector across malaria transmission zones in Senegal (2014–2017)

Malaria transmission zone	Facility type	Percentage of expected cases reported				Percentage of facilities reporting				Percentage of indicator values reported			
		2014	2015	2016	2017	2014	2015	2016	2017	2014	2015	2016	2017
**Low**	**Health posts**	90.2	94.0	94.8	97.3	84.2	91.3	95.7	97.8	83.6	91.0	95.4	97.7
	**Health centers**	93.2	91.8	94.7	99.2	86.7	90.8	95.7	99.7	86.7	90.8	95.7	99.7
	**Hospitals**	81.4	97.3	78.7	66.3	76.7	92.1	66.3	92.1	76.7	92.1	66.3	92.1
**Moderate**	**Health posts**	91.3	96.2	99.7	99.4	88.3	96.5	98.9	99.2	88.0	95.9	97.6	98.7
	**Health centers**	94.3	91.2	100.0	98.7	88.8	91.7	100.0	99.7	88.8	91.7	100.0	99.7
	**Hospitals**	100.0	95.7	80.8	74.5	100.0	88.0	85.2	78.7	100.0	88.0	85.2	78.7
**High**	**Health posts**	87.5	89.8	98.5	95.7	84.4	94.4	97.0	96.4	83.4	94.0	96.5	96.1
	**Health centers**	95.7	81.0	99.0	90.9	89.7	83.3	98.6	90.6	89.7	83.3	98.6	90.6
	**Hospitals**	N/A	100.0	88.6	94.1	N/A	100.0	87.5	81.9	N/A	100.0	87.5	81.9

## Discussion

This research, which assessed the quality of reporting of malaria data in Senegal during the first 4 years of DHIS2 implementation, identified key strengths of the reporting system. Our analyses focused on the data quality dimensions of completeness and internal consistency for three key malaria indicators. Overall, we find that public sector facilities in Senegal are well-represented in the DHIS2 and have generally achieved high levels of reporting completeness and internal consistency at national and subnational levels. This finding is consistent with the general improvements in reporting completeness that have been observed in the DHIS2 systems in multiple other countries [35, 37–39]. The progress in Senegal is likely attributable to longstanding efforts by the NMCP, the DSISS and their partners to improve reporting via the DHIS2 platform. Since the designation of the DHIS2 as the national RHIS in 2016, on an annual basis the NMCP has dedicated significant financial resources to support the implementation of the DHIS2 system [18]. Furthermore, the NMCP and DSISS enjoy support from international and local partners to strengthen the health system and improve data quality. With these partnerships and MoH support, the medical regions are able to conduct quarterly data review workshops and health districts are able to support data related activities such as trainings and supervision in individual facilities thus maintaining sustained focus on the importance of data quality [12, 18].

Despite the successes observed among public facilities in Senegal, only 15.3% of private facilities reported their data in the DHIS2 during the study period. This finding is consistent with previous studies elsewhere in Africa showing low representation of private facilities in DHIS2 [35, 37]. In the West African region, Senegal has a relatively large private health sector compared to its neighbors [27, 40]. Nonetheless, it is generally thought that the private for-profit sector in Senegal is minimally involved in malaria treatment and prevention activities with many facilities referring patients to public and not-for-profit private facilities where malaria services are provided free of charge [27]. In 2017, 20% of children who sought care for fever in the 2 weeks preceding the nationally representative Demographic and Health Survey (DHS) obtained treatment from private health facilities with 12% seeking care from pharmacies [41] most of which operate on a for-profit basis [27]. It thus stands to reason that ensuring broader coverage of the DHIS2 system within the private sector would increase the representativeness of reported data. This is particularly true for the Dakar region with over 80% of the private sector facilities [27].

Consistent with previous observations by others, we found that hospitals generally had lower levels of indicator completeness compared to health posts. Similarly, a recent study focusing on the quality of reporting of maternal and newborn care indicators in northern Nigeria found that the completeness of reported indicators was significantly lower in hospitals compared to primary care facilities [42]. In Senegal, the lower rates of indicator completeness among hospitals may be explained by the fact that unlike other facility types in the public sector, public hospitals are not directly supervised by health districts. Furthermore, whereas health posts and health centers submit their reports to the district where the data is first verified before entry into the DHIS2 system, hospitals typically enter the data directly into the DHIS2 thereby bypassing additional checks. Although hospitals presumably manage a small proportion of malaria cases in Senegal, with only 3.9% of febrile children seeking care in public hospitals [16], the NMCP, DSISS and their partners should nonetheless strive for stronger collaboration between hospital data teams and regional health management teams for data-related activities such as data quality reviews, feedback and supervision. This level of coordination is essential to optimize routine data for monitoring and surveillance [37, 38, 42] in addition to ensuring regional data completeness. Nonetheless, additional investigations are needed in order to identify the root causes of the lower reporting rate among hospitals compared to health posts and health centers.

Analyses examining differences in RHIS reporting completeness by season are seldom conducted. Whereas the average monthly percentage of facilities reporting did not differ by season, we observed a modest but statistically significant increase of 7.5% in the average percentage of district-level monthly cases reported during the rainy season compared to the dry season. Although small, this difference may have important programmatic implications particularly in the northern districts implementing pre-elimination strategies [12]. When malaria transmission is very low, an accurate count of passively detected malaria cases is continuously needed to plan effectively for case investigations and reactive focal testing [43]. Considering that a higher proportion of expected malaria cases was generally missed in the low transmission zone as compared to other zones, the NMCP and its partners should continue strengthening surveillance efforts particularly in the northern districts. In these settings, training, supervision and messaging interventions should be designed for data entry staff to encourage consistent data collection practices throughout the year. On the basis of the findings that completeness rates were generally lower for facilities in the high transmission zone regardless of facility type, we urge renewed focus on data quality reviews and encouraging facilities in this zone to increase DHIS2-based reporting. Given that the regions in this zone are largely rural with geographically remote areas, it is possible that weak telecommunications and unstable supply of electricity in the regions [44] could have impacted reporting. These issues have been previously identified as barriers to reporting in Senegal [45]. Nonetheless, additional studies are needed to identify facility-level characteristics that may help explain differences in reporting completeness observed across malaria transmission zones.

Analyses of reporting consistency over time showed that more confirmed malaria cases, suspected cases and tests conducted were reported in 2017 compared to the average of the three previous years. These findings are most likely explained by the steady increase in reporting completeness and more importantly by the adoption of an updated NMCP policy, beginning in 2015, of testing all febrile patients under the age of five with an RDT, regardless of any other signs or symptoms [46]. The expansion of the definition of a suspect case of malaria resulted in a 97% increase in the number of suspect cases, 102% increase in the number of tests conducted and an 85% increase in the number of confirmed cases from 2014 to 2015 [47]. It is also important to note that a remarkable change in rainfall levels occurred between 2014 (which was exceptionally dry) and 2015 (an exceptionally wet year) [48]. Increases in rainfall levels may result in ecological changes that favor the proliferation of mosquito breeding sites, which could in turn lead to increased transmission of malaria [49].

In this study, we combined outpatient and community-based malaria data reported for those health posts that were associated with health huts. Although the DHIS2 is configured such that health hut data can be reported separately from the health post data, determining which health posts were accurately linked to their associated health huts and the extent to which the health posts were reporting already combined data proved to be challenging. Furthermore, the DHIS2 is also configured such that DSDOM data may be reported separately. Given the high occurrence of blanks and zeros in these data, however, it was challenging to analyze these data. Taken together, these aspects precluded a detailed analyses comparing the quality of facility-based and community- based data in Senegal. Nonetheless, given that the country has a longstanding history of community-based management of malaria and other illnesses [22], we urge that future assessments of DHIS2 malaria data quality should evaluate community-based reporting.

A deeper examination of facility-level zero reporting practices is also warranted as it has been shown elsewhere in sub-Saharan Africa that some facilities fail to report “zero” values in the DHIS2 when there are no malaria clinical events captured [37, 50]. This leads to an inability to properly distinguish true missing values from “zero” values. Unsurprisingly, we found that zero values were more common during the dry season and in areas with lower transmission of malaria. This is to be expected since malaria-related events should become less frequent with decreasing transmission. Nonetheless, the extent to which missing DHIS2 indicator values reflect an absence of malaria-related events remains unclear in the Senegal context. Given the need for sensitive surveillance in the northern districts implementing pre-elimination activities, it is essential that training on consistent zero-reporting practices be strengthened among frontline staff.

### Limitations

This study had limitations that warrant further discussion. First, our data management process excluded 19.7% of the facilities configured in the DHIS2 at the time of the study. We thus only analyzed facilities that were expected to report in the DHIS2 and not necessarily all those that were required to report as dictated by MoH policy. In the absence of up-to-date annual facility master lists and inadequate information to determine when individual facilities were active, we excluded facilities that did not report for the 12 months of the calendar year. The exclusion of facilities may have the effect of overestimating the different measures of data quality considered in this paper. This is particularly important to remember when interpreting results from 2014 since most hospitals and facilities from 5 districts did not report in the DHIS2 system for the year, presumably due to lags in the implementation of the system.

Second, we excluded private health facilities from our analyses. Given the small proportion of private facilities actually reporting in the DHIS2 at the time of this analysis, we felt that any trends observed from those facilities would not be representative. A separate analysis is thus appropriate to investigate the quality of malaria reporting in the private sector.

Third, our assessment of the percentage of facilities represented in the DHIS2 relied on the master facility list used for the 2017 SPA survey. This master list resulted from the national facility census in 2016 which was the most recent by the time of this study. The facility count during enumeration activities may differ from the total count of facilities existing in Senegal in 2017. This difference may in turn affect the accuracy of our results. We also note that the registration of facilities from master facilities to the DHIS2 is a continuous process that may change over time. This is especially true for private facilities operating outside of direct MoH control that may be more challenging to enumerate and register to the DHIS2 [51]. We are unable to fully assess the effects of these changes in facility registration over time.

Fourth, our analyses do not address the accuracy nor the external consistency of DHIS2 malaria data in Senegal. The accuracy of routine data is typically determined by assessing the degree to which the data reported in the RHIS compares to data in facility-level registers [52]. The methods and resources required for this exercise were beyond the scope of the desk review of data quality that we sought to conduct. The external consistency of routine data measures the agreement of routine health data with a “gold standard,” usually defined to be survey data [13]. Although nationally representative DHS were conducted in Senegal throughout the study period, the indicators in our study do not lend themselves to valid comparisons to those available in the surveys. An analysis of the concordance between the malaria data reported in DHIS2 and those collected using the NMCP’s Excel spreadsheet would have improved the rigor of our data quality assessment. We were unable to access the NMCP data for a more detailed analysis. To support the planned efforts to integrate the NMCP system into DHIS2 [53], future assessments should include a comparison of malaria data from both sources.

Finally, the quasi-Poisson model used in our analyses assumes similar seasonality of malaria cases across all facilities considered. Ignoring the variation in seasonality for individual facilities increases the likelihood that the model may have produced poor fits for some facilities. Ultimately, this limitation likely had a limited impact on the overall results since we were interested in the average seasonality across all facilities.

Despite these limitations, this study enriches the literature by adding to the body of evidence showing increasing quality of DHIS2 data. Though the WHO recommends annual desk reviews of routine data quality, results from studies examining the quality of DHIS2 data are rarely published. By demonstrating the extent to which these data are complete and consistent, we hope that our findings may enable researchers and policy-makers to use malaria DHIS2 data with more confidence.

## Conclusion

The completeness and consistency of reporting of key malaria indicators in Senegal has improved since the implementation of the DHIS2 system in 2014. Nonetheless, we noted some shortcomings that will need to be addressed to harness DHIS2’s full potential. First, we identified many facilities configured in the DHIS2 that are inactive, some without clear documentation of when they started reporting in the DHIS2 and others without ownership information. Continuous maintenance of the DHIS2 system will be required to ease data use by analysts and health managers. Secondly, a large proportion of private sector facilities currently remains excluded from the DHIS2. This omission will continue to hamper the representativeness of DHIS2 data, particularly in urbanized areas, such as the Dakar region, where the private sector is most active. Strategies incentivizing DHIS2 uptake and reporting among private facilities should be explored.

The NMCP and the DSISS should ensure the sustainability of district-level data quality reviews and facility-level supervision. Maintaining feedback mechanisms from the central level to the facility will further emphasize the need for quality data. Furthermore, this may also increase levels of engagement towards producing and using quality data at all echelons of the health system.

Future assessments of DHIS2 malaria data should be more comprehensive and focus on additional malaria indicators such as those measuring rates of malaria hospitalizations, malaria treatment and preventive services, malaria-related commodities among others. Since the concept of data quality has more dimensions than were possible to address in our study, future studies should also examine the quality of DHIS2 reporting based on these other metrics. To support the efforts to integrate the NMCP Excel-based system into the DHIS2, it is critical that future studies compare the quality of malaria data produced across the two systems, in addition to the concordance between the data. Future assessments should also consider a detailed comparison of malaria data from facility-based and community-based reporting in DHIS2.

## Supplementary Information

**Additional file 1 **: **Supplementary Table 1**. Description of public facilities reporting malaria data in DHIS2 by year, facility type and transmission zone. **Supplementary Table 2**. Description of public health posts reporting community-based malaria data in DHIS2 by year. **Supplementary Table 3**. District-level zero reporting by season across examined indicators among public facilities.

## Data Availability

All data are publicly available through the national District Health Information Software 2 online database at https://senegal.dhis2.org/.

## Abbreviations

CNERS: Senegal National Ethical Committee for Health Research
DHIS2: District Health Information System Version 2
DHS: Demographic and Health Survey
DSISS: Division of Social and Health Information Systems
MOH: Ministry of Health
NMCP: National Malaria Control Program
RDT: Rapid diagnostic test
RHIS: Routine Health Information system
SPA: Service Provision Assessment survey
WHO: World Health Organization

## References

[1] Tatem AJ, Smith DL, Gething PW, Kabaria CW, Snow RW, Hay SI (2010). Ranking of elimination feasibility between malaria-endemic countries. Lancet (London, England).

[2] Cotter C, Sturrock HJ, Hsiang MS (2013). The changing epidemiology of malaria elimination: new strategies for new challenges. Lancet.

[3] World Health Organization. Global technical strategy for malaria 2016–2030. Geneva: World Health Organization; 2015.

[4] District Health Information System 2. 2020; https://www.dhis2.org. Accessed 12 June 2021.

[5] Kenya National Malaria Control Programme (2014). Malaria Surveillance Bulletin.

[6] Ghana National Malaria Control Programme. National malaria control surveillance 1st quarter bulletin. Accra; 2016.

[7] Tanzania National Malaria Control Programme (2018). Malaria surveillance bulletin: 3rd and 4th quarter 2017.

[8] Uganda National Malaria Control Programme (2016). Uganda malaria quarterly bulletin issue 14: April–June 2016.

[9] Idrissa SANE (2016). Utilisation à grande échelle du logiciel Dhis2 : Le Sénégal dans l’ère révolutionnaire de la gestion de l’information sanitaire.

[10] PMI. Annual revised funding tables for Senegal. 2014–2017; https://www.pmi.gov/resource-library/mops/. Accessed 12 June 2020.

[11] Global Fund (2016). Sustainably improve the health of the Senegalese populations through health systems strengthening (Grant: SEN-S-MOH).

[12] PMI (2017). President’s malaria initiative: Senegal malaria operational plan FY 2018.

[13] World Health Organization (2017). Data quality review module 2: desk review of data quality.

[14] Agence nationale de la statistique et de la démographie (ANSD) (2018). La population du Senegal en 2017.

[15] Ndiaye M, Faye B, Tine R (2012). Assessment of the molecular marker of plasmodium falciparum chloroquine resistance (Pfcrt) in Senegal after several years of chloroquine withdrawal. Am J Trop Med Hyg.

[16] DHS Program (2014). Enquete Demographique et de Sante Continue (EDS-Continue) Sénégal.

[17] Programme National De Lutte Contre Le Paludisme. Bulletin épidémiologique annuel 2017 du paludisme au Sénégal. Dakar; 2017.

[18] Programme National De Lutte Contre Le Paludisme. Plan strategique national de lutte contre le paludisme au senegal 2016–2020. Dakar; 2015.

[19] Niang M, Thiam LG, Sow A (2015). A molecular survey of acute febrile illnesses reveals plasmodium vivax infections in Kedougou, southeastern Senegal. Malar J.

[20] Littrell M, Sow GD, Ngom A (2013). Case investigation and reactive case detection for malaria elimination in northern Senegal. Malar J.

[21] DHS Program (2017). Senegal service provision assessment (SPA) survey.

[22] Gaye S, Kibler J, Ndiaye JL (2020). Proactive community case management in Senegal 2014–2016: a case study in maximizing the impact of community case management of malaria. Malar J.

[23] DHS Program (2014). Enquête Continue sur la Prestation des Services de Soins de Santé (ECPSS): 2014.

[24] DHS Program (2015). Enquête Continue sur la Prestation des Services de Soins de Santé (ECPSS): 2015.

[25] DHS Program (2016). Enquête Continue sur la Prestation des Services de Soins de Santé (ECPSS): 2016.

[26] DHS Program (2017). Enquête Continue sur la Prestation des Services de Soins de Santé (ECPSS): 2017.

[27] Brunner B, Barnes J, Carmona A (2016). Senegal private health sector assessment: selected health products and services.

[28] Evaluation Task Force of Roll Back Malaria’s Monitoring and Evaluation Reference Group (2019). Framework for evaluating national malaria programs in moderate- and LowTransmission settings.

[29] Francis D, Gasasira A, Kigozi R (2012). Health facility-based malaria surveillance: the effects of age, area of residence and diagnostics on test positivity rates. Malar J.

[30] Wedderburn RW (1974). Quasi-likelihood functions, generalized linear models, and the gauss—Newton method. Biometrika..

[31] Ver Hoef JM, Boveng PL (2007). Quasi-Poisson vs. negative binomial regression: how should we model overdispersed count data?. Ecology..

[32] Singh RB, Hales S, de Wet N, Raj R, Hearnden M, Weinstein P (2001). The influence of climate variation and change on diarrheal disease in the Pacific Islands. Environ Health Perspect.

[33] Peng RD, Dominici F, Louis TA (2006). Model choice in time series studies of air pollution and mortality. J R Stat Soc: Ser A (Statistics in Society).

[34] Gasparrini A, Armstrong B (2010). Time series analysis on the health effects of temperature: advancements and limitations. Environ Res.

[35] Nisingizwe MP, Iyer HS, Gashayija M (2014). Toward utilization of data for program management and evaluation: quality assessment of five years of health management information system data in Rwanda. Glob Health Action.

[36] StataCorp LLC (2015). Stata statistical software: release 14.

[37] Githinji S, Oyando R, Malinga J (2017). Completeness of malaria indicator data reporting via the district health information software 2 in Kenya, 2011–2015. Malar J.

[38] Kiberu VM, Matovu JK, Makumbi F, Kyozira C, Mukooyo E, Wanyenze RK (2014). Strengthening district-based health reporting through the district health management information software system: the Ugandan experience. BMC Med Inf Decis Making.

[39] O'Hagan R, Marx MA, Finnegan KE (2017). National Assessment of data quality and associated systems-level factors in Malawi. Glob Health, Sci Pract.

[40] Brunner B, Carmona A, Kouakou A, Dolo I, Revuz CU, Thierry MLK, Sessi. (2014). The private health sector in West Africa: six macro-level assessments.

[41] DHS Program (2017). Enquete Demographique et de Sante Continue (EDS-Continue) Sénégal.

[42] Bhattacharya AA, Umar N, Audu A (2019). Quality of routine facility data for monitoring priority maternal and newborn indicators in DHIS2: a case study from Gombe state, Nigeria. PLoS One.

[43] World Health Organization (2012). Disease surveillance for malaria control: an operational manual.

[44] Doran D, Fox A (2016). Operationalizing central place and central flow theory with mobile phone data. Ann Data Sci.

[45] Muhoza P, Saleem H, Faye A (2021). Key informant perspectives on the challenges and opportunities for using routine health data for decision-making in Senegal. BMC Health Serv Res.

[46] Thwing J, Ba F, Diaby A (2017). Assessment of the utility of a symptom-based algorithm for identifying febrile patients for malaria diagnostic testing in Senegal. Malar J.

[47] Programme National De Lutte Contre Le Paludisme. Bulletin épidémiologique annuel 2016 du paludisme au Sénégal. Dakar; 2016.

[48] Brandt M, Tappan G, Diouf AA, Beye G, Mbow C, Fensholt R (2017). Woody vegetation die off and regeneration in response to rainfall variability in the west African Sahel. Remote Sens.

[49] Mouchet J, Manguin S, Sircoulon J (1998). Evolution of malaria in Africa for the past 40 years: impact of climatic and human factors. J Am Mosq Control Assoc.

[50] Maina JK, Macharia PM, Ouma PO, Snow RW, Okiro EA (2017). Coverage of routine reporting on malaria parasitological testing in Kenya, 2015–2016. Glob Health Action.

[51] World Health Organization. Creating a master health facility list. World Heal Organ. 2013:1–49.

[52] World Health Organization (2017). Data quality review module 3: data verification and system assessment.

[53] PMI (2020). President’s Malaria Initiative: Senegal Malaria Operational Plan FY 2020.

