# Supplementary material for: A data quality assessment of the first four years of malaria reporting in the Senegal DHIS2, 2014–2017
Source: BMC Health Serv Res. 2022 Jan 2;22:18. doi: 10.1186/s12913-021-07364-6 (PMC8722300; doi:10.1186/s12913-021-07364-6)
Supplement: Supplementary file 1 — Additional file 1 : Supplementary Table 1. Description of public facilities reporting malaria data in DHIS2 by year, facility type and transmission zone. Supplementary Table 2. Description of public health posts reporting community-based malaria data in DHIS2 by year. Supplementary Table 3. District-level zero reporting by season across examined indicators among public facilities. [file 12913_2021_7364_MOESM1_ESM.docx]

**A data quality assessment of the first four years of malaria reporting in the Senegal DHIS2, 2014-2017**

# **Authors**

Pierre Muhoza*, PhD, Johns Hopkins Bloomberg School of Public Health, Department of International Health, 615 N. Wolfe Street, Baltimore MD 21205, Email: [pmuhoza1@jhmi.edu](about:blank)

Roger Tine, MD / PhD, *Département de Parasitologie, Centre de Recherche de Keur Socé, Faculté de Médecine, Université Cheikh Anta Diop de Dakar*, Dakar, Senegal, Email: [roger.tine@ucad.edu.sn](about:blank)

Adama Faye MD / PhD, *Institut de Santé et Développement*, *Université Cheikh Anta Diop de Dakar*, Dakar, Senegal, Email: [adamafaye94@gmail.com](adamafaye94@gmail.com%20)

Ibrahima Gaye, MS, *Institut de Santé et Développement*, *Université Cheikh Anta Diop de Dakar*, Dakar, Senegal, Email: [ibrahima-gaye@live.fr](ibrahima-gaye@live.fr%20)

Scott L. Zeger, PhD, Johns Hopkins Bloomberg School of Public Health, Department of Biostatistics, 615 N. Wolfe Street, Baltimore MD 21205, Email: [sz@jhu.edu](mailto:sz@jhu.edu)

Abdoulaye Diaw, MD, Direction de la Planification, de la Recherche et des Statistiques/ Division du Système d’Information Sanitaire et Sociale, Ministère de la Santé et de l'Action Sociale (MSAS), Dakar, Senegal, Email : <layejaw@yahoo.fr>

Alioune Badara Gueye, MD, Programme National de Lutte Contre le Paludisme, Ministère de la Santé et de l'Action Sociale (MSAS), Dakar, Senegal, Email : <badou_gueye@hotmail.com>

Almamy Malick Kante, PhD, Johns Hopkins Bloomberg School of Public Health, Department of International Health, 615 N. Wolfe Street, Baltimore MD 21205, Email: [akante1@jhu.edu](mailto:akante1@jhu.edu)

Andrea Ruff, PhD, Johns Hopkins Bloomberg School of Public Health, Department of International Health, 615 N. Wolfe Street, Baltimore MD 21205, Email: [aruff1@jhu.edu](mailto:aruff1@jhu.edu)

Melissa A. Marx, PhD, Johns Hopkins Bloomberg School of Public Health, Department of International Health, 615 N. Wolfe Street, Baltimore MD 21205, Email: [mmarx@jhu.edu](mailto:mmarx@jhu.edu)

Key words: DHIS2, routine health information systems, data quality, Senegal, malaria

**Supplementary table 1:** **Description of public facilities reporting malaria data in DHIS2 by year, facility type and transmission zone**

|  | **Facility type** | **Malaria transmission zone** | | |  |
| --- | --- | --- | --- | --- | --- |
|  |  | **Low n (%)** | **Moderate  n (%)** | **High n (%)** | **Total n (%)** |
| **2014** | **Health posts** | 550 (52.9) | 240 (23.1) | 248 (24.0) | 1,038 (100.0) |
|  | **Health centers** | 44 (52.4) | 23 (27.4) | 17 (20.2) | 84 (100.0) |
|  | **Hospitals** | 5 (71.4) | 2 (28.6) | 0 (0.0) | 7 (100.0) |
| **2015** | **Health posts** | 679 (55.9) | 264 (21.7) | 271 (22.3) | 1,214 (100.0) |
|  | **Health centers** | 55 (53.4) | 25 (24.3) | 23 (22.3) | 103 (100.0) |
|  | **Hospitals** | 20 (57.1) | 9 (25.7) | 6 (17.2) | 35 (100.0) |
| **2016** | **Health posts** | 711 (55.9) | 280 (22.0) | 281 (22.1) | 1,272 (100.0) |
|  | **Health centers** | 54 (52.9) | 25 (24.6) | 23 (22.5) | 102 (100.0) |
|  | **Hospitals** | 21 (58.3) | 9 (25.0) | 6 (16.7) | 36 (100.0) |
| **2017** | **Health posts** | 733 (55.6) | 295 (22.4) | 290 (22.0) | 1,318 (100.0) |
|  | **Health centers** | 54 (52.9) | 25 (24.6) | 23 (22.5) | 102 (100.0) |
|  | **Hospitals** | 20 (57.1) | 9 (25.7) | 6 (17.2) | 35 (100.0) |

**Supplementary Table 2 Description of public health posts reporting community-based malaria data in DHIS2 by year**

| **Facility type** | **2014 n (%)** | **2015 n (%)** | **2016 n (%)** | **2017 n (%)** |
| --- | --- | --- | --- | --- |
| **Health posts reporting community data for all 12 months** | 700 (67.4) | 875 (72.1) | 1,097 (86.2) | 1,189 (90.2) |
| **Health posts reporting community data for some but not all 12 months** | 331 (31.9) | 330 (27.2) | 161(12.7) | 127 (9.6) |
| **Health posts failing to report any community data for all 12 months** | 7 (0.7) | 9 (0.7) | 14 (1.1) | 2 (0.2) |
| **Total public health posts reporting in the DHIS2** | **1,038** **(100.0)** | **1,214** **(100.0)** | **1,272** **(100.0)** | **1,318 (100.0)** |

**Supplementary Table 3 District-level zero reporting by season across examined indicators among public facilities**

|  | **2014**** | **2015** | **2016** | **2017** |
| --- | --- | --- | --- | --- |
| **Average percent zeros reported during the dry season (SE)** | 35.3 (2.4) | 38.9 (1.5) | 31.7 (1.3) | 33.2 (1.0) |
| **Average percent zeros reported during the rainy season (SE)** | 27.8 (2.1) | 21.9 (1.5) | 22.5 (1.3) | 24.7 (1.2) |
| **Difference (SE)**  **(P value)***** | 7.5 (3.2) 0.02 | 17.0 (2.1) <0.001 | 9.2 (1.9) <0.001 | 8.5 (1.6) <0.001 |
| ^**^ Analyses do not include data from 5 districts from the regions of Fatick (moderate transmission zone) and Matam (low transmission zone) that did not report malaria data in the DHIS2 throughout 2014 ***P values result from a two-sample t-test assuming equal variances comparing district percentage of zeros reported during rainy versus dry seasons. | | | | |
